# Deployment of a Second Victim Peer Support Program: A Replication Study

**DOI:** 10.1097/pq9.0000000000000031

**Published:** 2017-06-21

**Authors:** Jenna Merandi, Nancy Liao, Dorcas Lewe, Shelly Morvay, Barb Stewart, Charline Catt, Susan D. Scott

**Affiliations:** From the * Nationwide Children’s Hospital, Columbus, Ohio; † The Ohio State University College of Medicine, Nationwide Children’s Hospital, Columbus, Ohio; ‡ Quality Improvement Services, Nationwide Children’s Hospital, Columbus, Ohio; § Patient Care Services, Nationwide Children’s Hospital, Columbus, Ohio; ¶ University of Missouri Health Care, Columbia, Mo.

## Abstract

**Background:**

The second victim phenomenon occurs when health-care providers experience significant professional distress (compassion dissatisfaction, burnout, secondary traumatic stress) and psychological distress (shame, anxiety, and depression) as a result of medical errors or adverse patient outcomes. Few hospitals have institution-wide systems in place to assist employees through the recovery process.

**Methods:**

At Nationwide Children’s Hospital (NCH), a peer-based support program called “YOU Matter” was executed and spread hospital-wide. The program emulated the framework and execution strategy designed by University of Missouri Health Care’s (MUHC) “forYOU” Team. Strategic elements of the program’s structure were reviewed and adapted for NCH with system-wide deployment and enhancement to include electronic peer support reporting. This article summarizes program implementation, management, and sustainment over the past 2 years.

**Results:**

By following University of Missouri Health Care’s model, we successfully deployed an institution-wide second victim program. Since the November 2013 initiation, we have documented 232 peer and 21 group encounters. High-risk clinical areas for second victimization at NCH included the emergency department (ED), pediatric intensive care unit (PICU), cardiothoracic intensive care unit (CTICU), and pharmacy department. Registered nurses (RNs) and licensed practical nurses (LPNs) have had the highest number of encounters necessitating second victim support (32%). Supported staff reported improved emotional state and improved return-to-work metrics.

**Conclusions:**

An organization’s culture of patient safety can be enhanced by ensuring staff psychological safety. Programs like “YOU Matter” and the “forYOU” Team are essential building blocks to improve the overall safety culture and quality of care. Implementation of “YOU Matter” at NCH validates the MUHC program and demonstrates its generalizability to other health-care institutions.

## BACKGROUND

Since the publication of the Institute of Medicine’s *To Err is Human: Building a Safer Health System*,^1^ a paradigm shift in medical error reporting has occurred.^2–5^ There is some indication that patient safety has improved.^6^ However, the support systems for health-care workers involved in medical errors are lacking.^7–9^ These second victims of unanticipated clinical events suffer emotionally and are traumatized by the events.^9–16^ Researchers estimate that 10–40% of health-care professionals have been second victims.^9,12–16^ Affected individuals may suffer from guilt, depression, sleep disturbance, anxiety, or even suicidal ideation.^17–19^ Although the vast majority of hospitals rely on employee assistance programs or pastoral care for providing “just-in-time” clinician support after adverse events, these services are often underutilized, and the suffering provider carries the burden of the adverse outcome.^10,11^

At Nationwide Children’s Hospital (NCH), the need for a comprehensive system-wide support system for second victims became apparent when unit leaders asked for the development of a second victim program. These leaders had learned about a system-wide support network at University of Missouri Health Care (MUHC) called the forYOU Team. The forYOU Team harnesses existing resources within health-care systems to address unmet needs of clinicians suffering as second victims.^20^

In an MUHC study, 30% (268/898) of responding health-care professionals reported second victim symptoms as a result of a patient safety event. Approximately 15% (40/269) reported seriously contemplating leaving their profession.^12^ The NCH pharmacy leaders replicated the survey and found similar findings among their staff. With a response rate of 67% (121/181), results revealed that 28% (34/121) of respondents suffered anxiety and sleep disturbance following an adverse event, 10% (12/121) contemplated leaving the institution, and 3% (4/121) considered leaving the pharmacy profession.^13^ As the NCH survey mirrored that of MUHC, the NCH staff decided to collaborate with MUHC patient safety researchers to initiate a similar second victim support program starting in 2012. This article describes the collaboration with MUHC researchers and the replication of the forYOU program.

## METHODS

### Setting

NCH is a free-standing pediatric, academic institution with over 10,000 employees, approximately 1,200 medical staff, approximately 17,000 inpatient admissions and more than 1 million patient contacts per year. NCH has a robust voluntary adverse event reporting system and a strong culture of patient safety.^5,6^ It is one of the founding members of the Solutions for Patient Safety and was the first pediatric institution to post its serious safety event online (see http://www.nationwidechildrens.org/serious-safety-event-rate-sser).

### Multidisciplinary Team Development

**Fig. 1.**
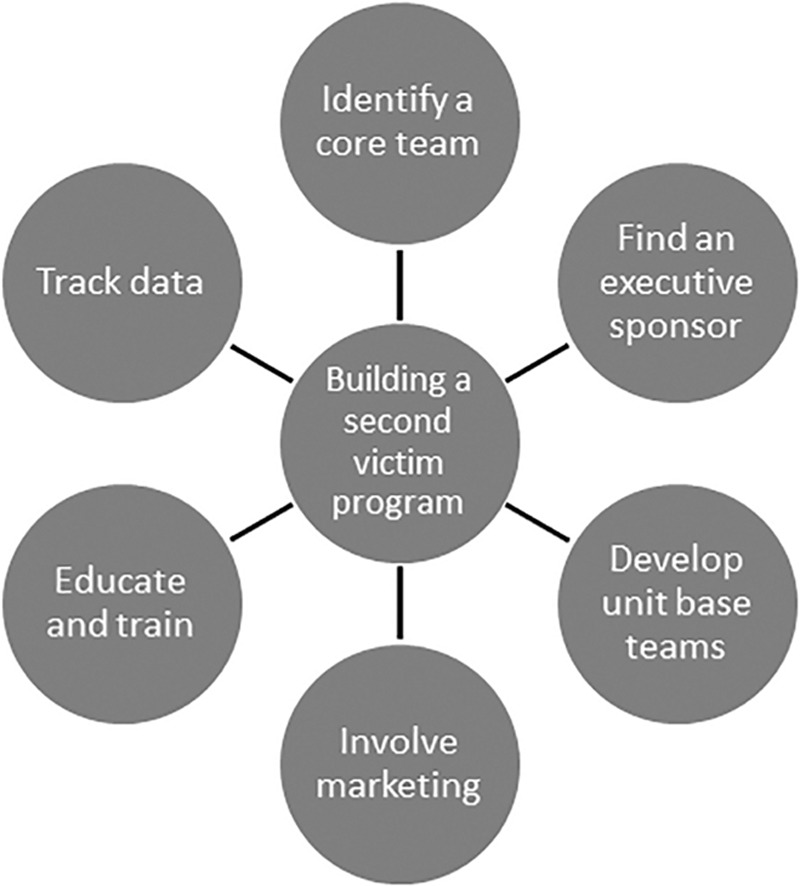
Listed are the six key components when initiating a second victim program.

In July 2013, we reached out to MUHC researchers to discuss strategies for development of a hospital-wide second victim support structure. Six components of team design identified were (1) identify a core steering team, (2) identify an executive sponsor, (3) develop unit-based teams, (4) develop team branding/marketing, (5) educate and train peer supporters, and (6) track data to ensure effectiveness (Fig. 1). We formed a multidisciplinary steering committee to implement the second victim program. The aim of the group was to increase awareness of the second victim phenomenon with an ultimate goal of implementing an institution-wide, peer-based, support system known locally as YOU Matter. An executive sponsor, program director, and administrative coordinator were identified to assist with overseeing program development/implementation as recommended by MUHC researchers. In the spirit of collaboration, the MUHC forYOU Team researchers shared training information, documents, marketing tools, policies, and procedures with NCH counterparts with the understanding that modification to meet particular NCH needs might be necessary.

Program development began by obtaining the support and involvement of key NCH leaders, including hospital executives, members of quality improvement/safety teams, and the legal department.^21,22^ The vice president of Patient Care Services (C.C.) agreed to serve as executive sponsor of the YOU Matter program. Director of Quality Improvement Services (A.R.) acted as a supportive resource throughout implementation. The executive sponsor and director of Quality Improvement Services served as liaisons to other executives, provided guidance and leadership, and exercised administrative oversight. The second victim’s mission aligned perfectly with one of the hospital’s strategic quality initiatives, Treat Me With Respect.^23^ Consequently, the YOU Matter program received financial support that was instrumental in the success of the program.

**Fig. 2.**
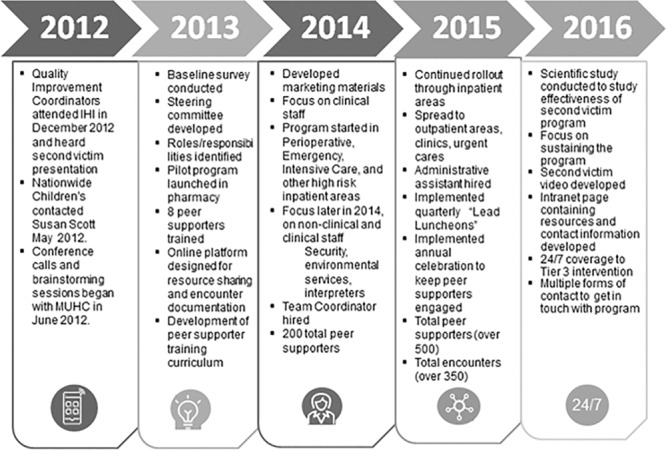
Second victim program timeline at NCH.

In November 2013, an initial demonstration pilot study among NCH pharmacy staff was conducted by Krzan et al.^13^ This successful pilot study supported the hospital-wide expansion of the NCH second victim program, first to the emergency department and subsequently to the perioperative department, intensive care unit’s (ICU), and surgical units in March 2014. The program was spread throughout all inpatient units as well as urgent cares, outpatient primary care clinics, and ambulatory specialty clinics. Timeline for program implementation is outlined in Figure 2.

### Program Marketing

We developed marketing materials (brochures, handouts, identification badge quick reference cards, and digital quality boards) Digital quality boards visible in staff lounges and conference rooms described the second victim phenomenon and promoted the new program. These various methods of marketing the program increased staff awareness and desire to become involved.

Outreach occurred initially with unit leaders then expanded to all employees. Inpatient clinical leaders were educated on the second victim phenomenon and were asked to nominate individuals who could serve as unit program champions. Desired characteristics of the unit champions included strong leadership skills, trustworthiness, effective communication skills, and personal second victim experience. Once confirmed, the leads committed to training and to oversee other peer supporters within their unit/department.

### Employee Education and Training

NCH collaborated with MUHC to establish the basic framework of education and peer supporter training. Although many similarities existed between how NCH and MUHC conducted their training sessions, NCH made adjustments to accommodate staffing needs. For example, NCH modified training time from 8 hours to 4 hours. This change eliminated the duplicate content of second victim personal stories, decrease time spent on role play and abbreviated session breaks (Table 1). NCH added additional training in the areas of electronic documentation, legal responsibilities, and coping mechanisms for the nonclinical staff. By structuring education and data collection materials directly from MUHC, the NCH steering committee implemented the program within a short period (Table 1). The team was operational within 6 months of concentrated effort as compared with MUHC which took over 2 years. To become a peer supporter, an individual completes a 4-hour workshop that included education on the second victim phenomenon and its natural history,^24^ an overview of existing literature, necessary skills for responding to second victims, provision of referrals and escalation of care, and legal/confidentiality considerations (Table 2).

**Table 1.**
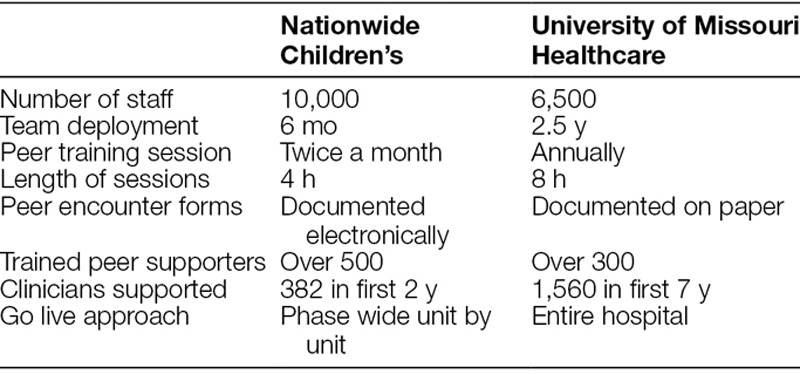
Comparisons between NCH Hospital and MUHC Second Victim Programs

**Table 2.**
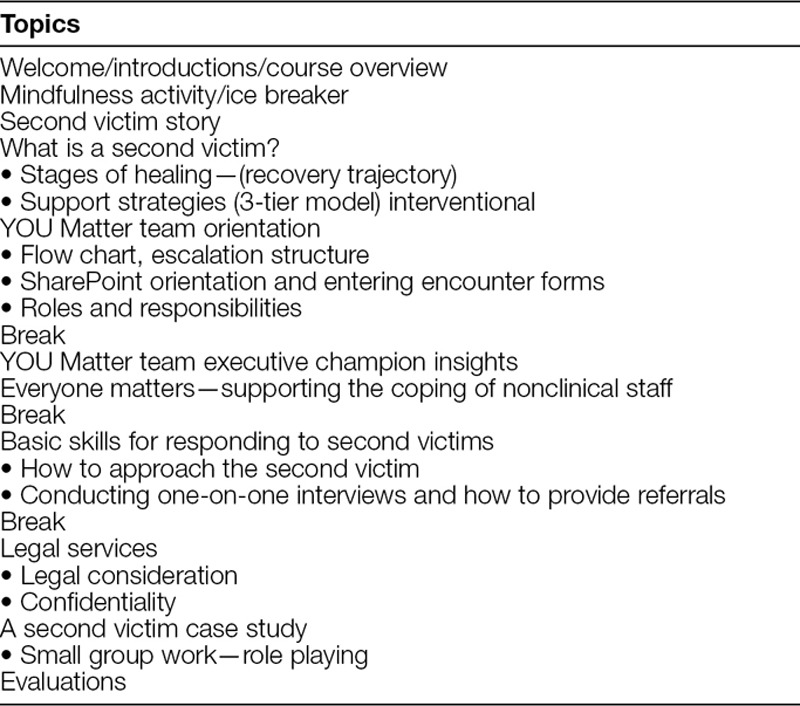
Nationwide Children’s Training Program Schedule

### Scott Three-Tiered Interventional Model of Support

The YOU Matter program uses the Scott Three-Tiered Interventional Model of Support for Second Victims,^12^ as follows:

- Tier 1—local unit/department support, providing one-on-one reassurance to second victims.
- Tier 2 consists of trained peer supporters, the patient safety team, and risk management activation if the second victim requires further assistance.
- Tier 3 results in expedited referral to ensure availability of professional support/guidance as needed (employee assistance program, chaplain, social work, clinical psychologist, and so on).

Members of the multidisciplinary steering committee provided tier 1 education to the entire institution through presentations regarding ways to identify a second victim, providing essential support, and referral processes when higher levels of support are warranted. Tier 2 support consisted of trained peer supporters. The goal for each unit was to have 10% of staff trained as peer supporters with all shifts and disciplines represented. This degree of participation allowed for 24/7 availability of YOU Matter team members.

Peer supporters received identification badge extenders for easy recognition as a peer supporter.

Tier 3 support included a team of chaplains, social workers, and clinical psychologists. A clinical psychologist provided training on responding to second victims and served as a team mentor. Tier 3 support also incorporated existing Employee Assistance Program (EAP) resources.

The YOU Matter program’s interventional model of support mirrors MUHC’s structure. Peer supporters provide first-line support to second victims and report to assigned leaders within various clinical areas. The leaders are capable of escalating issues to the core steering committee and tier 3 resources as needed.

**Fig. 3.**
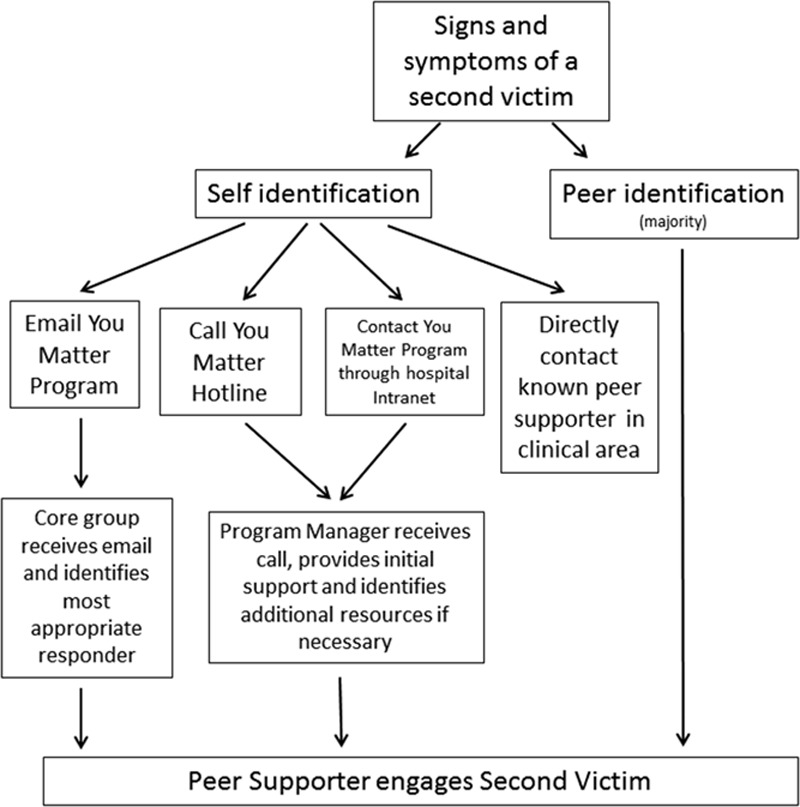
Ways to activate peer support.

As with its MUHC counterpart, staff may activate the YOU Matter program support through the following 4 mechanisms (Fig. 3):

1. Direct contact with peer supporters as identified by the YOU Matter badge
2. Utilization of the YOU Matter 24/7 hotline
3. Usage of the Second Victim Support e-mail group
4. Direct contact with core team members as identified on intranet site.

### Electronic Documentation

As the second victim program continued to grow, the need for efficient communication and documentation became apparent. A “Second Victim SharePoint site” was created serving as a central, electronic portal, to manage the program, share information, and document encounters (SharePoint, Microsoft Corporation, Redmond, Wash.).^13^ The primary purpose of documentation is to quantify frequency and types of second victim encounters.

We established security permissions to ensure confidentiality of the second victim program and to limit access to the YOU Matter Program SharePoint site. Access was granted at various security levels depending on the individual’s role. Once staff completed initial training, the permission level was changed to “peer supporter” so they could document peer/group encounters. Individuals were able to access only peer and group encounter forms that they initiated. The program director had access to all encounters and tracked necessity for further interventions.

Electronic peer/group encounter forms were adopted from MUHC with nonidentifiable personal health information data captured and converted to the online platform (SharePoint, Microsoft Corporation).^13^ Data collected from the electronic portal were analyzed to better understand the second victim experience on NCH staff and to implement system changes to enhance support.

The second victim SharePoint site provides real-time dashboard metrics with the following information: number of peer supporters, disciplines of peer supporters, the number of peer/group encounters, and encounter locations.

## RESULTS

**Fig. 4.**
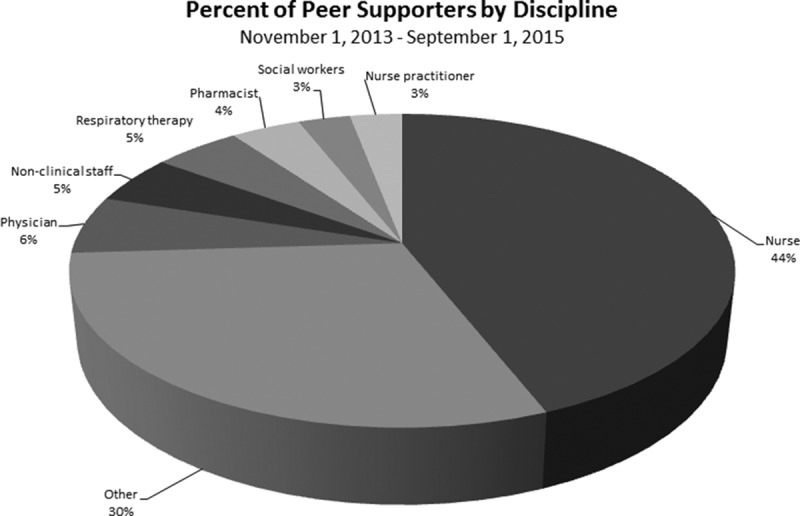
Percentage of peer encounters by discipline.

**Fig. 5.**
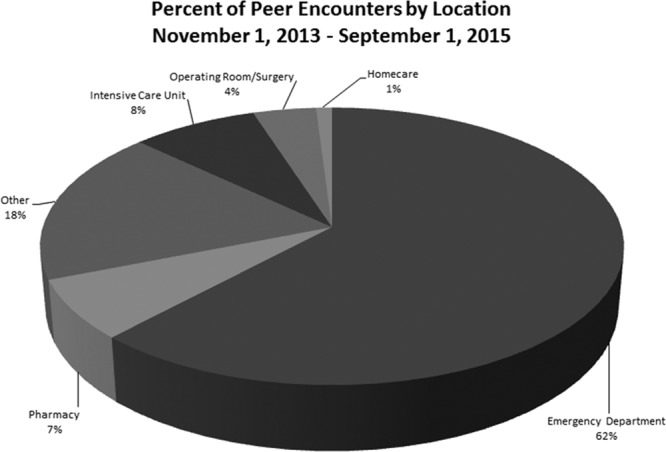
Peer encounters by location.

Since the November 2013 team deployment, there are over 300 peer supporters trained, 232 peer and 21 documented group encounters, and 30 leaders identified. Demographically, nurses comprise 44% of peer supporters, and other staff (multiple disciplines including clinical and nonclinical personnel) include 30% of peer supporters (Fig. 4). Of the 232 documented peer encounters, 62% occurred in ED, and 8% in pediatric intensive care unit and cardiothoracic intensive care unit (Fig. 5). Additional areas with a higher number of encounters include the pharmacy department and other inpatient hospital units.

**Fig. 6.**
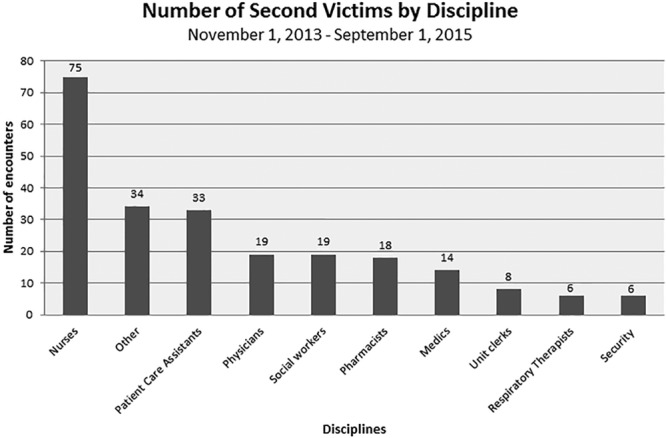
Number of second victims by discipline.

Nurses have the highest number of encounters documented (75 of total encounters of 232) as receiving second victim support, followed by other staff (34), which included certified nurse anesthetists, interpreters, suture technicians, child life specialists, physical/occupational therapists, and medical assistants. Patient care assistants were involved in 33 encounters followed by, physicians (19), social workers (19), and pharmacists (18; Fig. 6).

**Fig. 7.**
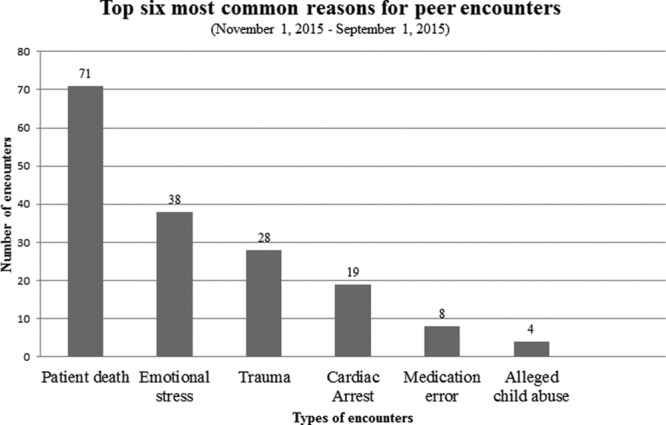
Reasons for peer encounters.

Since program conception, 6 common reasons for peer encounters have been patient death, emotional stress, trauma, cardiac arrest, medication error, and alleged child abuse cases (Fig. 7). The most common group encounters included the death of a colleague and an unexpected patient demise.

## DISCUSSION

This article describes replication of MUHC’s forYOU Team at a large pediatric institution. Key elements to the success of this project were the establishment of an effective working relationship between 2 independent health-care entities and the willingness to “test” key team infrastructure components within the context of a unique health-care entity. This successful partnership permitted NCH to implement a successful second victim support program. Using MUHC’s established model for clinician support, NCH deployed an operational program in 6 short months. The primary infrastructure components replicated included team design, the Scott Three-Tiered Model of Second Victim Support, training materials, and program monitoring tools. Through the fruitful efforts of the YOU Matter team, organizational recognition of clinicians in distress and the social support of second victims has steadily increased. Implementation of the team has enhanced the overall culture of sensitivity and psychological safety at NCH.

We have replicated critical aspects of the MUHC second victim support program. Using the MUHC framework for the design of a support team and implementation strategies were considered essential to a timely program launch. Second, hospital administration support was necessary for identification of an executive sponsor being strategic. Without the support from hospital administration and guidance from the legal department, a peer support program would be difficult to operationalize successfully. Third, the creation of a multidisciplinary core team was critical. Peer-to-peer education was more readily received. Recruitment was also more successful on a peer-to-peer basis. Peer recommendations were the foundation of the program. The core team represented all hospital staff from inception to be appropriately impactful. Fourth, program promotion involved the institution’s marketing department in helping brand the team concept and increase awareness at every level of the institution.

Lastly, documentation of events was essential to track program impact and identify targeted areas in need of additional support. Constructing an electronic tracking system via SharePoint allowed team leadership to collect valuable data regarding the incidence of second victims, types of encounters, types of emotional support provided, and disciplines that were involved.

The justification for a second victim program is often not concrete and lacks obvious financial incentives for hospital systems. Delays may occur due to these constraints when implementing a program. Investing time in education and raising awareness of the second victim phenomenon among hospital administration is helpful to augment program initiation.

The YOU Matter program had a disproportionate representation of peer supporters across disciplines. The data show that approximately 6% of employees at NCH received training as peer supporters (which include clinical and nonclinical staff). Nurses may be overrepresented because of their workday availability and involvement in hospital committees. For future team development, we endorse strategic recruitment efforts that yield representative distribution representing all staff. The Nationwide Children’s goal was to incorporate at least 5–10% of staff trained as peer supporters for each area/unit. Having a variety of individuals trained as peer supporters on various shifts allows for more opportunities to access a peer supporter if needed.

Interestingly, as we disseminated the program throughout the institution, and staff awareness increased regarding the second victim phenomenon, the team received anecdotal reports from nonclinical staff who were adversely impacted by traumatic clinical events. Although the definition of second victims primarily involves clinician, we recognized the need to include nonclinical staff as well. As with the MUHC experience, the YOU Matter team expanded the program to include security guards, interpreters, unit clerks, environmental services staff, and other nonclinical employees.^25,26^

The YOU Matter data demonstrate that higher numbers of second victims exist in high-risk areas and units with child abuse and undesirable patient outcomes. The ED and ICUs had the largest number of encounters documented. This trend is not observed in perioperative or surgical areas perhaps due to poorer documentation or fewer trained peer supporters compared with the ED and ICU. Although peer supporters may provide second victim support, documented encounters of the emotional support provided may be lacking.

Future directions for the YOU Matter program include recruiting new members to more accurately reflect staff distribution and to study the effectiveness of the peer support model. Previously, MUHC’s forYOU Team demonstrated that the most efficient kind of support comes from peers.^12^

Though survey results from employees appear to find the program beneficial, it will be important to define further the effects of the YOU Matter interventions. We are currently undergoing a study to quantify program effectiveness.

## CONCLUSIONS

When an error occurs, the health-care provider can be overlooked and is left to handle the emotional repercussions alone. A culture of safety for patients is enhanced by a program that it is equally focused on the psychological safety of staff.

Peer support is the preferred method of support by medical personnel; however, initiating a large-scale, second victim rapid response team can be a daunting undertaking for any health-care facility.^22^ Following the successful MUHC model proved to be an efficient and practical approach to NCH team design and deployment. Health-care facilities without emotional support for staff need to consider the implementation of a second victim peer support program and evaluate its effectiveness.^27^

The NCH’s system-wide intervention has demonstrated that second victims occur across the health-care continuum with an increased prevalence in areas of high patient acuity and death. Second victims report improved emotion state and benefit from the program. We feel that rapidly and widely accessible support is critical to an overall culture of safety for any health-care institution. Based on the NCH experience, the MUHC clinician support model is transferrable to other health-care entities with minor modifications and is an efficient way to develop similar future staff support initiatives within health-care facilities.

## ACKNOWLEDGMENTS

We thank Anamarie Rayburn, Director of Quality Improvement Services; Lois Stepney, LISW, Critical Incident Stress Coordinator and YOU Matter Team Coordinator; Laura Hirschinger, forYOU Team, and Franklin Wall, Administrative Coordinator, YOU Matter Program.

## DISCLOSURE

The authors have no financial interest to declare in relation to the content of this article.
